# Altered pharmacological effects of adrenergic agonists during hypothermia

**DOI:** 10.1186/s13049-016-0339-8

**Published:** 2016-12-05

**Authors:** Erik Sveberg Dietrichs, Georg Sager, Torkjel Tveita

**Affiliations:** 1 Anesthesia and Critical Care Research Group, Department of Clinical Medicine, UiT, The Arctic University of Norway, 9037 Tromsø, Norway; 2 Department of Research and Education, Norwegian Air Ambulance Foundation, 1441 Drøbak, Norway; 3 Experimental and Clinical Pharmacology, Department of medical biology, UiT, The Arctic University of Norway, 9037 Tromsø, Norway; 4 Division of Surgical Medicine and Intensive Care, University Hospital of North Norway, 9038 Tromsø, Norway

**Keywords:** Hypothermia, Cooling, Rewarming, Rearming shock, Pharmacology, Cardiovascular dysfunction, Adrenergic drugs, Inotropic, Vasopressor

## Abstract

Rewarming from accidental hypothermia is often complicated by hypothermia-induced cardiac dysfunction, calling for immediate pharmacologic intervention. Studies show that although cardiac pharmacologic support is applied when rewarming these patients, a lack of updated treatment recommendations exist. Mainly due to lack of clinical and experimental data, neither of the international guidelines includes information about pharmacologic cardiac support at temperatures below 30 °C. However, core temperature of accidental hypothermia patients is often reduced below 30 °C. Few human studies exploring effects of adrenergic drugs during hypothermia have been published, and therefore prevailing information is collected from pre-clinical studies. The most prominent finding in these studies is an apparent depressive effect of adrenaline on cardiac function when used in doses which elevate cardiac output during normothermia. Also noradrenaline and isoprenaline largely lacked positive cardiac effects during hypothermia, while dopamine is a more promising drug for supporting cardiac function during rewarming. Data and information from these studies are in support of the prevailing notion; not to use adrenergic drugs at core temperatures below 30 °C.

## Background

Rewarming victims of accidental hypothermia is often complicated by hypothermia-induced cardiac dysfunction. In its fulminant form this condition is described as rewarming shock; an acute heart failure with a progressive fall in cardiac output (CO) where the patient terminates in a sudden and intractable fall in blood pressure [1]. This serious complication to clinical therapy adds to the virtually unchanged low survival rate of accidental hypothermia over the last decades [2, 3]. Cardiac supportive therapy has to be instituted during rewarming in an attempt to prevent the imminent cardiovascular collapse. Inotropic drugs, i.e. drugs that enhance force of cardiac contraction [4] could provide such pharmacologic support, but current guidelines do not support this view. Both the American Heart Association and the European Resuscitation Council advise against using drugs like adrenaline below 30 °C [5, 6]. Studies investigating patient treatment do however report that inotropic drugs are administered during rewarming in 47–66% of patients [7, 8]. Only about 10% of patients with acute heart failure caused by disease or events other than hypothermia receive the same treatment [9]. This lack of consensus-based guidelines on cardiac inotropic support in hypothermic patients cause confusion, even within the British health care system [10]. Importance of finding optimal treatment for these patients is manifest from that survival is possible after extreme exposure to hypothermia. Case-reports show that early resuscitation enables survival after cooling to 13.7 °C [11] or cardiac arrest close to 7 h [12], but mortality rate is still reported at 30% [2]. Seeking evidence-based pharmacologic treatment options, we have explored the literature for pre-clinical and clinical studies on use of inotropic drugs during hypothermia and rewarming, with interesting findings. Most studies, including several from our group, focus on adrenergic receptor agonists. The information provided, presents a valuable insight in hypothermia-induced changes in cardiovascular pharmacology, laying foundation for development of new treatment strategies and guidelines in a patient group exposed to lethal cardiac complications during rewarming.

## Methods

The aim of this paper was to describe effects of adrenergic agonists during hypothermia. Relevant publications were found through literature search, using PubMed (Medline) and Google Scholar search engines. Experimental and clinical studies included in this narrative review were selected according to their relevance by the authors, who all have a special interest in hypothermia and pharmacology. Reference lists of included papers were studied to discover publications that were not detected through use of search engines. Additional articles describing pathophysiology of hypothermia, treatment guidelines and general knowledge on adrenergic agonists and receptor function, were included for background information.

### Adrenergic receptors (Table 1)

Extracellular binding of adrenergic receptor agonists facilitates initiation of intracellular processes through G-protein coupled signalling. In 1948, Ahlquist described how the adrenergic receptors are divided into two main groups [13], named α- and β-receptors. Subgroups have later been identified and separated the receptors into α_1–2_ and β_1–3_, which have a broad variety of effects, among them hemodynamic. The β_1_-receptor is considered most important for inotropic effect and is also more numerous in the mammalian heart (75%) than β_2_ and β_3_ [14]. β_1_-stimulation enhances heart muscle contraction, increase heart rate and enhance relaxation of myocardial tissue [15]. The effect of β_1_-agonists is mediated through stimulation of adenylyl cyclase, which elevates cyclic AMP (cAMP). This activates protein kinase A (PKA), which phosphorylates several proteins. Phosphorylation of sarcolemmal L-type calcium channels gives increased calcium influx, thus enabling contraction, while phosphorylation of cardiac troponin I enhances myocardial relaxation [14]. β_2_-stimulation most importantly gives vasodilation due to smooth muscle relaxation [16]. Both α_1_- and α_2_- receptors are divided into three subgroups. Stimulation of all α_1_-receptors will in general induce smooth muscle contraction, which gives vasoconstriction [17]. The α_2_-receptor subgroups have differing abilities, among them both vasodilation and vasoconstriction [18]. Cardiovascular effects of adrenergic stimulation are also transmitted through dopamine receptors by direct effect on smooth muscle, giving vasodilation mediated by D_1_-like receptors and indirectly by D_2_-like receptors. Several dopamine receptor subtypes are located in the human heart, namely D_1,_ D_2_, D_4_, and D_5_ [19], but stimulation of these receptors does not have a pronounced effect on cardiac contractility in rats [20]. Adrenergic receptor agonists have varying affinity for β-, α- and dopamine-receptors and their subgroups, explaining their distinct properties.

**Table 1:** Adrenergic receptors

	α-receptor	β-receptor	Dopamine-receptor
Subgroups	α_1_, α_2_	β_1_, β_2_, β_3_	D_1_, D_2_, D_3_, D_4_, D_5_
Mechanism of action	G-protein coupled receptors	G-protein coupled receptors	G-protein coupled receptors
Molecular effects	α_1_: Activation of PLC, IP3 mediated intracellular calcium increase α_2_: Decreased cAMP production	Increase of cAMP, protein phosphorylation (β_1_, β_2_, β_3_), intracellular calcium increase (β_1_) or decrease (β_2_)	D_1_, D_5_: Increase of cAMP D_2_, D_3_, D_4_: Decreased cAMP production
Hemodynamic effect	α_1_: Vasoconstriction↑ α_2_: Mixed (vasoconstriction or vasodilation)	β_1_: Heart rate↑, contractility↑ β_2_: Vasodilation↑	D_1_, D_5_: Vasodilation↑ D_2_, D_3_, D_4:_: Vasoconstriction↓
Dominant location	α_1_: Smooth muscle α_2_: Central nervous system	β_1_: Cardiac tissue β_2_: Smooth muscle β_3_: Adipose tissue	D_1_, D_2_, D_3_, D_4_, D_5:_ Central nervous system

### β_1_-receptor function during hypothermia

As the β_1_-receptor is considered most important for providing inotropic effect in normothermic conditions [14] studies on administering adrenergic drugs to ameliorate rewarming shock have also targeted this receptor. In a study from our lab we have reported a 4-fold increase of in vivo cardiac cAMP content during β_1_-receptor stimulation at 15 °C, showing that β_1_-receptor function is not depressed at low temperatures. This was confirmed by an in vitro 9-fold increase of β_1_-receptor sensitivity in isolated rat cardiomyocytes cooled to 15 °C [21]. Such hypothermia-induced in vitro β_1_-receptor super-sensitivity was also described in isolated heart preparations from guinea pig [22, 23] and rabbit hearts at 22 °C [24]. Results from intact (in vivo) animal experiments studying effects of adrenergic receptor-ligands and -blockers indicate that both β_1_- and β_2_-receptor function is maintained during cooling to 28–30 °C, but that response to agonist binding is depressed by cooling below 30 °C [25–27]. The relationship between an apparent increase in β-receptor sensitivity and decreased inotropic effect below a core temperature of 30 °C is uncertain. Mann [28] found excessive cAMP levels, seen during β_1_-receptor stimulation in hypothermic rats [21], to be cardiotoxic, through initiating un-physiological increase of cytosolic calcium levels, mediated by increased phosphorylation of L-type calcium channels [28]. Hypothermia-induced calcium overload is also known to take place in response to prolonged hypothermia in rats per se [29, 30] and an additional increased calcium load in response to a pharmacologic stimulation might explain lacking effect of β_1_-agonists during hypothermia. Another observed effect of temperature reduction in rats is reduced myocardial calcium sensitivity due to hypothermia-induced elevation of PKA-mediated phosphorylation of Ser 23/24 at cardiac troponin I [31]. Increased levels of cAMP will increase such PKA-mediated Ser 23/24 phosphorylation and give a negative inotropic effect. Such phosphorylation will also enhance cardiac relaxation [32], which is normalized during rewarming from hypothermia as diastolic function is restored [33]. Thus, favorable or harmful effects of β_1_-agonists administration in hypothermic subjects appears associated to inotropic rather than lusitropic properties, as hypothermia-induced cardiac dysfunction is an isolated impairment of systolic function [1].

The aforementioned studies do however show differences in species and experimental conditions and use several β_1_-receptor agonists with varying properties, including α-receptor agonism that promotes vasoconstriction. A resulting increase of systemic vascular resistance (SVR) cause a pronounced negative effect on cardiac function in rats during hypothermia [21, 27, 34, 35]. For assessment of clinical properties of these drugs, it is therefore necessary to evaluate their individual pharmacologic effects when used during hypothermia.

### Adrenergic receptor agonists (Table 2, Fig. 1)

#### Adrenaline

Adrenaline will enhance cardiac contraction and heart rate and either decrease (low-dose) or increase (high-dose) SVR in normothermic conditions [36], conducted by loss of β-adrenergic selectivity at low doses with increasing α-stimulation at higher doses.

**Table 2:** β_1_-receptor agonists

	Adrenaline	Noradrenaline	Isoprenaline	Dobutamine	Dopamine
Species	Rat, dog	Cat, dog,	Rat, dog, rabbit, guinea pig	Dog, pig, rabbit, guinea pig	Dog, pig
Dosage (in vivo)	0.4 μg/kg/min – 4.2 μg/kg/min	0.2 μg/kg/min – 5.0 μg/kg/min	5.7 ng/kg/min – 1 μg/kg (bolus)	2.0 μg/kg/min – 30 μg/kg/min	2.0 μg/kg/min – 30 μg/kg/min
Administration	i.v. (in vivo studies), Retrograd coronary perfusion (in vitro studies)	i.v. (in vivo studies)	i.v. (in vivo studies), Retrograd coronary perfusion or in preparation solution (in vitro studies)	i.v. (in vivo studies), Retrograd coronary perfusion or in preparation solution (in vitro studies)	i.v. (in vivo studies)
Target temperature	12 °C–28 °C	28 °C–30 °C	20 °C–28 °C	22 °C–31 °C	25 °C–30 °C
Cardiac effect (hypothermia)	Elevated CO (low dose) [27, 34, 38]. Depressed CO (high dose) [21, 27, 34, 35, 37]. Negative inotropic effects (in vitro) [41, 42].	Increased contractile force [26, 45]. Depressed CO [40].	No effect on CO [50, 51]. Negative or depressed inotropic effect (in vitro) [52, 53]. Positive inotropic effects (in vitro) [22, 24, 54].	Elevated CO [38, 57]. Increased contraction velocity (in vitro) [24]. Reduced or depressed inotropic effect (in vitro) [56].	Elevated CO or positive inotropic effects [57, 61, 63]. No effect on CO [62, 63].

**Fig. 1:**
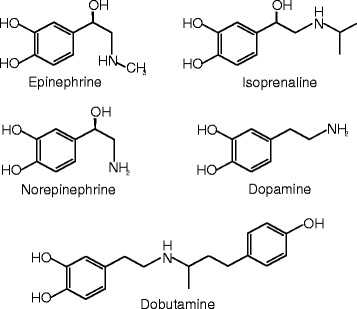
Molecular structure of adrenaline, noradrenaline, isoprenaline, dopamine and dobutamine

##### Adrenaline during hypothermia

Pharmacodynamic effects of adrenaline do not seem to be independent of temperature changes. Rubinstein found that doses inducing vasodilation in normothermic dogs would give increased SVR during hypothermia and stated that the inotropic effect of adrenaline is reduced at 25 °C [37]. A similar study on rats conducted in our lab, showed that a high dose of adrenaline (1.25 μg/min) increased stroke volume (SV) and CO in normothermic animals. When an equal dose was administered during rewarming from 15 °C however, SV and CO were unaffected. In contrast, a low dose (0.125 μg/min) adrenaline, which induced vasodilation during normothermic conditions, failed to reduce SVR or mean arterial pressure (MAP) during rewarming, but led to an elevated CO [34]. The positive effect of low-dose adrenaline during hypothermia has also been reported from experiments using dogs [38]. We found the same dose–response relationship during cooling, where 0.125 μg/min but not 1.25 μg/min adrenaline gave positive cardiac effects during cooling to 28 °C. After rewarming, only rats that had received saline during cooling showed pre-hypothermic hemodynamic responses to adrenaline [27]. An additional study on rats from our lab showed that 1 μg/min of adrenaline given during cooling caused a maintained depression of cardiac function during rewarming [35]. These results indicate that hypothermia has a severe impact on cardiac inotropic effects mediated by the β_1_-receptor pathway, as β_1_-adrenergic stimulation during hypothermia also has a negative impact on inotropic effect of β_1_-agonists after rewarming. The same phenomenon is also observed in a feline model of hypothermia and rewarming [39, 40]. From a combined in vitro and in vivo study in our lab, we showed that this hypothermia-induced reduction of inotropic effect via β_1_-receptor stimulation is seen in the presence of in vivo and in vitro β_1_-receptor super-sensitivity. Both increased β_1_-receptor binding and elevated cAMP levels were seen during administration of β_1_-receptor ligands in hypothermic conditions, as compared to normothermia. This study indicates that the detrimental effects of adrenaline during hypothermia is a consequence of adrenaline-induced increase in SVR via peripheral vascular α-receptor stimulation [21]. Failure of low doses of adrenaline to reduce MAP during hypothermia [34], further implies the presence of increased α-receptor agonism, or reduced effect of β_2_-receptor agonism in hypothermia.

Altogether, the present hypothermia-induced alteration of pharmacodynamic effects and an expected narrowed therapeutic window of adrenaline, advocate against the use of this drug during hypothermia. This assumptions gain support by data from in vitro experiments. In isolated rat hearts, both SV and CO were depressed by adrenaline at 28 °C [41], while at 12 °C positive inotropic effects were absent [42]. Interestingly, Schiffmann et al. demonstrated that in normothermic rat hearts, the presence of additionally added calcium would potentiate the inotropic effects of adrenaline. In the hypothermic heart however, increased calcium concentrations [41] mediated a depressive effect of adrenaline on SV and CO. Thus, the negative effects of adrenaline at low temperatures could be a consequence of hypothermia-induced calcium overload, reported in vitro [43], as well as in vivo [29, 30].

#### Noradrenaline

Noradrenaline has high affinity for α-receptors in concert with β_1_-receptor affinity. Infusion of noradrenaline therefore leads both to vasoconstriction of arterioles and a positive inotropic effect [44].

##### Noradrenaline during hypothermia

Cotten et al. demonstrated positive inotropic effect of noradrenaline in both normothermic controls and hypothermic (30 °C) dogs [26]. They further described that although this effect was positive, the inotropic effect of noradrenaline was reduced by hypothermia [45]. In cats subjected to moderate hypothermia and rewarming, noradrenaline had a negative effect on CO during hypothermia. The ability of noradrenaline to induce vasoconstriction did however appear intact during cooling, with consistent dose-related increase in MAP [40]. Intact α-receptor function is also apparent during noradrenaline infusion in humans cooled on cardiopulmonary bypass to 28–32 °C. In these patients MAP increased significantly with noradrenaline [46]. This can be explained by intact α_1_-receptor function during a wide range of temperatures, as demonstrated in sheep arteries where vascular response to noradrenaline abolished first when cooling to 5 °C [47]. The sensitivity of α-receptors was even found to be increased in human skin artery preparations cooled to 24 °C [48].

#### Isoprenaline

Isoprenaline is a non-selective β-receptor agonist, lacking α-agonist effect. On this background studies looking exclusively at β-receptor stimulation often use isoprenaline as a model drug. During normothermic conditions, isoprenaline will increase CO and decrease SVR through β_1_- and β_2_-receptor stimulation [49].

##### Isoprenaline during hypothermia

Conflicting results exist of pharmacologic effects of isoprenaline at low temperatures, especially when comparing results obtained from in vitro interventional studies of cardiac tissue from different species. Lauri et al. studied in vivo hemodynamic effects of isoprenaline before, during and after severe hypothermia (25 °C) in dogs. Isoprenaline had no positive inotropic effect during hypothermia, but a significant decrease in SVR indicated at least partly intact β_2_-receptor response to stimulation [50], also seen in man at 28–32 °C [46]. Inotropic effects of increasing isoprenaline doses have been investigated in our intact rat model [51] at normothermia and during cooling to 24 °C. We reported that the dose-related positive inotropic response (increase in SV and CO) to isoprenaline at 37 °C were lost during cooling to 24 °C except for the highest dose (20 ng/min). This alteration in response to β-receptor stimulation also remained after rewarming as only the highest dose of isoprenaline managed to elevate SV above baseline [51]. In vitro studies have shown depressed β_1_-mediated inotropic effect of isoprenaline in rat left atrial preparations at 28 and 20 °C, compared to at 35 °C [52]. This finding finds support in another study reporting reduced inotropic response to both isoprenaline and adrenaline in hypothermic rabbit atria at 23 °C [53]. In contrast, in isolated guinea pig hearts cooled to 27 °C isoprenaline still increased the contractility parameter LV dp/dt_max_, this was accompanied by a similar increase in heart rate, also mediated by β_1_-receptor stimulation [54]. Further, an experiment on isolated atria from guinea pig showed increased inotropic effects of isoprenaline at 25 °C [22]. Sustained ability of adrenaline and isoprenaline to increase contraction amplitude and rate has also been reported when cooling rabbit hearts to 22 °C [24].

#### Dobutamine

Dobutamine predominantly binds to β_1_-receptors and has a weak effect on β_2_- and α-receptors. Thus, administering dobutamine in normothermic conditions elevates CO [55].

##### Dobutamine during hypothermia

The inotropic effects of dobutamine shows temperature dependency when tested in in vitro guinea pig trabecula. Rieg et al. [56] therefore concluded that elevating cAMP through β_1_-receptor stimulation for providing inotropic support in hypothermic hearts is not an optimal strategy. In isolated rabbit hearts however, dobutamine infusion increased contraction velocity at 22 °C [24]. Increased cardiac output was also observed in response to dobutamine infusion during hypothermia (30 °C) in pigs [57]. Administering dobutamine in an intact dog model during reperfusion following 60 min global ischemia at 28 °C also showed promising cardiac effects, with increased SV during rewarming using cardio-pulmonary bypass [38].

#### Dopamine

Like adrenaline, noradrenaline, isoprenaline and dobutamine, dopamine is a catecholamine, giving dose-dependent stimulation of α- and β-receptors and giving a dose-dependent positive inotropic effect in normothermia [58]. Different from selective adrenergic agonists, dopamine also exerts its inotropic and vasoactive effects through stimulation of dopamine receptors [59].

##### Dopamine during hypothermia

The use of dopamine as a vasopressor is recommended in the Up To Date accidental hypothermia guidelines [60]. This is supported by a better cardiovascular recovery in dopamine-treated dogs, after core cooling to 25 °C and subsequent rewarming [61]. Likewise, positive inotropic effects of dopamine were found in pigs core cooled to 30 °C [57]. However, in pigs surface cooled to 32 °C [62] and 25 °C [63] dopamine did not elevate CO. In the latter study, which was conducted in our lab [63], dopamine infusion at 25 °C gave a four-fold increase in plasma concentration compared to normothermia. In difference from other β-adrenergic drugs, cardiovascular responses of dopamine were restored during rewarming to 30 °C [63]. It is therefore apparent from different animal models that dopamine supports cardiac function during rewarming, but it is uncertain whether these positive effects are present during hypothermia below 30 °C. Based on these findings, dopamine is the preferred drug for giving cardiac support during rewarming in the Northern-Norwegian guidelines for accidental hypothermia [64].

## Conclusion

### Pharmacodynamics

A lack of human studies evaluating cardiovascular effects of adrenergic drugs during hypothermia exists [65]. In the meantime such information can be collected from preclinical experimental studies. This information provide important insight on the effects of pharmacologic interventions already applied in the hypothermic patient. It is apparent that inotropic response to β-adrenergic stimulation seems to be depressed during severe hypothermia. This response is also depressed after rewarming in animals that have received such drugs during hypothermia [51]. In hypothermic animals, adrenaline increases SVR [21]. Studies on dobutamine administration indicates a positive effect, but these studies are carried out at temperatures around 30 °C [38, 57]. Advice of the current guidelines, not to use adrenergic drugs like adrenaline below 30 °C is supported by these preclinical observations, but recommendations for inotropic support in severe accidental hypothermic (>30 °C) patients are needed. Dopamine does not seem to have the same detrimental effects on cardiovascular function in severe accidental hypothermia as adrenaline, even in high plasma concentrations [63], but lack effect before patients are rewarmed to higher temperatures.

### Pharmacokinetics

Knowledge about pharmacokinetic effects of adrenergic drugs during hypothermia is limited. However, all pharmacokinetic processes are temperature-dependent; including absorption, distribution and elimination (metabolism and excretion). In general, lowered temperatures slow all these processes down. The time to reach distribution equilibrium will be lengthened and metabolism in the liver and active excretion in the kidneys reduced. Thus half-life (T_1/2_) of active substances is increased at low temperatures [66]. In humans, it is known that the cytochrome P450 enzyme system is affected by hypothermia. Tortorici [67] found that this resulted in a 7–22% reduced clearance per degree below 37 °C of opiates, barbiturates, benzodiazepines and neuromuscular blockers. Hypothermia does also induce changes in pharmacokinetics of adrenergic drugs. Reduced catechol-O-methyl transferase activity has therefore been suggested to explain a hypothermia-induced hypersensitivity to β-adrenoceptor agonists [24]. Increased T_1/2_ of adrenaline might therefore have contributed to elevated stimulation of β-adrenoceptors in a recent study reporting increased cAMP levels during 5 min adrenaline administration in hypothermia [21], as the normothermic T_1/2_ of adrenaline is 2 min [68]. Reduced enzymatic breakdown of cAMP through reduced phosphodiesterase 3 activity, or reduced extracellular release of cAMP as observed in cold fibroblasts [69], might also have boosted adrenaline-mediated cAMP increase in hypothermic hearts [21]. Hypothermia-induced increase in T_1/2_ is apparent for other catecholamines. At 25 °C in anesthetised pigs, we found that dopamine infusion yielded plasma concentrations 4 times higher than during normothermia. The half-life of dopamine was doubled at this temperature and returned to normothermic values first at 35 °C during rewarming [63]. The high concentrations of dopamine were however not associated with any negative hemodynamic effects.

Apart from temperature–dependent pharmacologic changes in ligand-receptor kinetics, changes in temperature also exert significant alterations in other determinants of cardiac function, which may limit the expected pharmacologic effects achieved at normothermia. During cooling, studies on isolated papillary muscle show a positive inotropic effect of hypothermia per se [70]. In the intact pig however, cooling induce a reduction of cardiac contractile function and SV [33]. Lewis and colleagues showed that the inotropic effect of increasing heart rate during normothermic conditions in man, is lost at a core temperature of 33 °C [71], independent of pharmacologic interventions. Consequently, hypothermia-induced changes in physiology, not related to ligand-receptor kinetics, could also be involved in altered pharmacodynamics of β-adrenoceptor agonists during hypothermic conditions. Important determinants of blood flow, like blood viscosity, are affected already at moderate hypothermia [72]. Thus, lack of ability of the cold blood to increase flow may be part of the challenging task to provide positive inotropic, pharmacologic support during hypothermia. The apparent depressed function of β_1_-receptor agonists to provide inotropic effect in vivo below 30 °C, might therefore be multifactorial. Updated guidelines on treatment of hypothermic patients depend on further studies exploring physiological effects of hypothermia and rewarming, as well as broader knowledge on the hypothermia-induced changes on pharmacodynamic and pharmacokinetic effects of drugs applied in clinical practice.

### Clinical implications

Findings in the reviewed literature indicate that negative or lacking effects of adrenergic drugs during hypothermia appears to be of multifactorial origin. Our findings from a majority of pre-clinical studies therefore advocate that drugs providing adrenergic receptor agonism should be used carefully during hypothermia and rewarming. Such information is highly relevant in the clinical setting. Reports show that pharmacological interventions are being used to provide cardiovascular support in a large proportion of patients during rewarming from accidental hypothermia [7, 8]. Hypothermia is also used as a therapeutic measure. Comatose survivors of cardiac arrest are often cooled to temperatures between 32–36 °C for cerebral protection [73]. More than 50% of this patient group are in need of inotropic support to facilitate adequate circulation [74]. Cooling and rewarming of patients down to, and occasionally below 20 °C is also used for cerebral protection during procedures like aortic arch surgery [73]. Providing optimal pharmacological, cardiovascular support in hypothermic patients therefore seems essential, both in therapeutic hypothermia, and when aiming to improve a high mortality rate associated with accidental hypothermia [2]. In pigs, dopamine appear a safe way to provide inotropic support, but lack effect at lower temperatures. Further, experimental studies have explored effects of inotropic pathways like PDE3 inhibition and calcium sensitizing, drugs that avoid the G-protein coupled adrenergic receptors. These experiments show promising results [75–79] on cardiovascular function, both during cooling and rewarming. However, information from such pre-clinical studies should be interpreted with care, when translated to a clinical, human setting. Aiming to provide better treatment, we call for further studies on physiology, therapeutic interventions and careful evaluation of inotropic drugs, used in hypothermic patients.

## Abbreviations

cAMP: Cyclic AMP
CO: Cardiac output
MAP: Mean arterial pressure
PKA: Protein kinase A
SV: Stroke volume
SVR: Systemic vascular resistance
T_1/2_: Half-life

## References

[1] Tveita T (2000). Rewarming from hypothermia. Newer aspects on the pathophysiology of rewarming shock. Int J Circumpolar Health.

[2] van der Ploeg G-J, Goslings JC, Walpoth BH, Bierens JJLM (2010). Accidental hypothermia: rewarming treatments, complications and outcomes from one university medical centre. Resuscitation.

[3] Maclean D, Maclean E-SD, Emslie-Smith (1977). Accidental hypothermia.

[4] Furnival CM, Linden RJ, Snow HM (1970). Inotropic changes in the left ventricle: the effect of changes in heart rate, aortic pressure and end-diastolic pressure. J Physiol.

[5] Vanden Hoek TL, Morrison LJ, Shuster M, Donnino M, Sinz E, Lavonas EJ (2010). Part 12: cardiac arrest in special situations: 2010 American Heart Association Guidelines for cardiopulmonary resuscitation and emergency cardiovascular care. Circulation.

[6] Truhlář A, Deakin CD, Soar J, Khalifa GEA, Alfonzo A, Bierens JJLM (2015). European resuscitation council guidelines for resuscitation 2015: section 4. Cardiac arrest in special circumstances. Resuscitation.

[7] Kornberger E, Schwarz B, Lindner KH, Mair P (1999). Forced air surface rewarming in patients with severe accidental hypothermia. Resuscitation.

[8] Vassal T, Benoit-Gonin B, Carrat F, Guidet B, Maury E, Offenstadt G (2001). Severe accidental hypothermia treated in an ICU: prognosis and outcome. Chest.

[9] Abraham WT, Adams KF, Fonarow GC, Costanzo MR, Berkowitz RL, LeJemtel TH (2005). In-hospital mortality in patients with acute decompensated heart failure requiring intravenous vasoactive medications: an analysis from the Acute Decompensated Heart Failure National Registry (ADHERE). J Am Coll Cardiol.

[10] Saraswatula A, Cornwell L, Latifi S (2008). Inconsistencies in the guidelines: use of adrenaline in paediatric cardiac arrest with hypothermia. Resuscitation.

[11] Gilbert M, Busund R, Skagseth A, Nilsen PA, Solbø JP (2000). Resuscitation from accidental hypothermia of 13.7 degrees C with circulatory arrest. Lancet.

[12] Kosinski S, Darocha T, Jarosz A, Migiel L, Zelias A, Marcinkowski W, et al. The longest persisting ventricular fibrillation with an excellent outcome – 6 h 45 min cardiac arrest. Resuscitation. 2016. 10.1016/j.resuscitation.2016.05.022 27283064

[13] Ahlquist RP (1948). A study of the adrenotropic receptors. Am J Physiol.

[14] Bers DM. Excitation-Contraction Coupling and Cardiac Contractile Force. Dordrecht: Springer; 2001. https://books.google.no/books?id=0p8AqZP7D5UC&pg=PA203&dq=Excitation-Contraction+Coupling+and+Cardiac+Contractile+Force+publisher+location&hl=no&sa=X&ved=0ahUKEwjp9tid5tPQAhUoYZoKHZKPB5gQ6AEIJDAB#v=onepage&q=Excitation-Contraction%20Coupling%20and%20Cardiac%20Contractile%20Force%20publisher%20location&f=false.

[15] Rohrer DK, Desai KH, Jasper JR, Stevens ME, Regula DP, Barsh GS (1996). Targeted disruption of the mouse beta1-adrenergic receptor gene: developmental and cardiovascular effects. Proc Natl Acad Sci U S A.

[16] Chruscinski AJ, Rohrer DK, Schauble E, Desai KH, Bernstein D, Kobilka BK (1999). Targeted disruption of the beta2 adrenergic receptor gene. J Biol Chem.

[17] Woodman OL, Vatner SF (1987). Coronary vasoconstriction mediated by alpha 1- and alpha 2-adrenoceptors in conscious dogs. Am J Physiol.

[18] Kable JW, Murrin LC, Bylund DB (2000). *In vivo* gene modification elucidates subtype-specific functions of alpha(2)-adrenergic receptors. J Pharmacol Exp Ther.

[19] Cavallotti C, Mancone M, Bruzzone P, Sabbatini M, Mignini F (2010). Dopamine receptor subtypes in the native human heart. Heart Vessels.

[20] Polakowski JS, Segreti JA, Cox BF, Hsieh GC, Kolasa T, Moreland RB (2004). Effects of selective dopamine receptor subtype agonists on cardiac contractility and regional haemodynamics in rats. Clin Exp Pharmacol Physiol.

[21] Dietrichs ES, Schanche T, Kondratiev T, Gaustad SE, Sager G, Tveita T (2015). Negative inotropic effects of epinephrine in the presence of increased β-adrenoceptor sensitivity during hypothermia in a rat model. Cryobiology.

[22] Chess-Williams RG, Broadley KJ, Duncan C (1984). A fundamental temperature-dependent difference between beta-adrenoceptor agonists and antagonists. Life Sci.

[23] Williams RG, Broadley KJ (1982). Responses mediated via beta 1, but not beta 2-adrenoceptors, exhibit hypothermia-induced supersensitivity. Life Sci.

[24] Riishede L, Nielsen-Kudsk F (1990). Myocardial effects of adrenaline, isoprenaline and dobutamine at hypothermic conditions. Pharmacol Toxicol.

[25] Melnikov AL, Løkebø JE, Helgesen KG, Lathrop DA (1997). Influence of hypothermia on the cardiac effects of propranolol observed in isolated rat atria. Gen Pharmacol.

[26] Cotten MV, Logan ME, Moore JI (1967). Relationships among cardiac inotropic responses to norepinephrine and cardiac and blood concentrations of H3-norepinephrine during hypothermia. J Pharmacol Exp Ther.

[27] Tveita T, Sieck GC (2011). The physiologic responses to epinephrine during cooling and after rewarming *in vivo*. Crit Care.

[28] Mann DL, Kent RL, Parsons B, Cooper G (1992). Adrenergic effects on the biology of the adult mammalian cardiocyte. Circulation.

[29] Kondratiev TV, Wold RM, Aasum E, Tveita T (2008). Myocardial mechanical dysfunction and calcium overload following rewarming from experimental hypothermia *in vivo*. Cryobiology.

[30] Wold RM, Kondratiev T, Tveita T (2013). Myocardial calcium overload during graded hypothermia and after rewarming in an *in vivo* rat model. Acta Physiol.

[31] Han YS, Tveita T, Prakash YS, Sieck GC (2010). Mechanisms underlying hypothermia-induced cardiac contractile dysfunction. Am J Physiol Heart Circ Physiol.

[32] Li L, Desantiago J, Chu G, Kranias EG, Bers DM. Phosphorylation of phospholamban and troponin I in beta-adrenergic-induced acceleration of cardiac relaxation. Am J Physiol Heart Circ Physiol. 2000;278:H769–79. 10.1152/ajpheart.2000.278.3.H769 10710345

[33] Filseth OM, How O-J, Kondratiev T, Gamst TM, Tveita T (2010). Post-hypothermic cardiac left ventricular systolic dysfunction after rewarming in an intact pig model. Crit Care.

[34] Kondratiev TV, Myhre ESP, Simonsen Ø, Nymark T-B, Tveita T (2006). Cardiovascular effects of epinephrine during rewarming from hypothermia in an intact animal model. J Appl Physiol.

[35] Kondratiev TV, Tveita T (2006). Effects of sympathetic stimulation during cooling on hypothermic as well as posthypothermic hemodynamic function. Can J Physiol Pharmacol.

[36] Rang HP, Dale MM, Flower RJ, Ritter JM, Henderson G (2011). Rang & Dale’s pharmacology.

[37] Rubinstein EH (1961). Vascular responses to adrenaline, noradrenaline and angiotensin in hypothermic dogs. Acta Physiol Lat Am.

[38] Sunamori M, Ozeki M, Okamura T, Amano J, Suzuki A (1985). Effects of catecholamines on myocardial viability in early reperfusion following hypothermic global ischemia in dogs--comparison between epinephrine and dobutamine. Jpn J Surg.

[39] Weiss SJ, Muniz A, Ernst AA, Lippton HL, Nick TG (2000). The effect of prior hypothermia on the physiological response to norepinephrine. Resuscitation.

[40] Weiss SJ, Muniz A, Ernst AA, Lippton HL (1998). The physiological response to norepinephrine during hypothermia and rewarming. Resuscitation.

[41] Schiffmann H, Gleiss J, von Hirscheydt A, Schröder T, Kahles H, Hellige G (2001). Effects of epinephrine on the myocardial performance and haemodynamics of the isolated rat heart during moderate hypothermia--importance of calcium homeostasis. Resuscitation.

[42] Nayler WG, Wright JE, Howells J. Effect of Epinephrine on the Mechanical and Phosphorylase Activity of Normo-and Hypothermic Hearts. Circ Res. 1963;13:199–206. 10.1161/01.res.13.3.199 14061808

[43] Aasum E (1997). Stimulation of carbohydrate metabolism reduces hypothermia-induced calcium load in fatty acid-perfused rat hearts. J Mol Cell Cardiol.

[44] Kirkendol PL, Woodbury RA (1972). Hemodynamic effects of infused norepinephrine in dogs on cardiopulmonary bypass. J Pharmacol Exp Ther.

[45] Moore JI, Cotten MV (1967). Influence of norepinephrine and ouabain of cardiac muscle mechanics during hypothermia. J Pharmacol Exp Ther.

[46] Baraka A, Haroun S, Baroody M, Nawfal M, Sibai A (1989). Action of adrenergic agonists on resistance ν capacitance vessels during cardiopulmonary bypass. J Cardiothorac Anesth.

[47] Keatinge WR (1964). Mechanism of adrenergic stimulation of mammalian arteries and its failure at low temperatures. J Physiol.

[48] Gómez B, Borbujo J, García-Villalón AL, Nava-Hernández E, Valle J, García JL (1991). Alpha 1- and alpha 2-adrenergic response in human isolated skin arteries during cooling. Gen Pharmacol.

[49] Beregovich J, Reicher-Reiss H, Grishman A (1972). Haemodynamic effects of isoprenaline in acute myocardial infarction. Br Heart J.

[50] Lauri T (1996). Cardiovascular responses to beta-stimulation with isoproterenol in deep hypothermia. J Appl Physiol.

[51] Han Y-S, Tveita T, Kondratiev TV, Prakash YS, Sieck GC (2008). Changes in cardiovascular β-adrenoceptor responses during hypothermia. Cryobiology.

[52] Melnikov AL, Løkebø JE, Lathrop DA, Helgesen KG (1996). Alteration of the cardiac effects of isoproterenol and propranolol by hypothermia in isolated rat atrium. Gen Pharmacol.

[53] Omar SA, Hammad D, Varma S (1979). Reduced beta adrenergic responsiveness in isolated rabbit atria during hypothermia. Indian J Physiol Pharmacol.

[54] Nakae Y, Fujita S, Namiki A (2001). Isoproterenol enhances myofilament Ca(2+) sensitivity during hypothermia in isolated guinea pig beating hearts. Anesth Analg.

[55] Stoner JD, Bolen JL, Harrison DC (1977). Comparison of dobutamine and dopamine in treatment of severe heart failure. Heart.

[56] Rieg AD, Schroth SC, Grottke O, Hein M, Ackermann D, Rossaint R (2009). Influence of temperature on the positive inotropic effect of levosimendan, dobutamine and milrinone. Eur J Anaesthesiol.

[57] Oung CM, English M, Chiu RC, Hinchey EJ (1992). Effects of hypothermia on hemodynamic responses to dopamine and dobutamine. J Trauma.

[58] Löllgen H, Drexler H (1990). Use of inotropes in the critical care setting. Crit Care Med.

[59] Contreras F (2002). Dopamine, hypertension and obesity. Int Congr Ser.

[60] Mechem CC, Danzl DF. Accidental hypothermia in adults [Internet]. uptodate.com. 2012 [cited 2013 Feb 1]. Available from: http://www.uptodate.com/contents/accidental-hypothermia-in-adults?source=search_result&search=accidental+hypothermia&selectedTitle=1%7E150. Accessed 1 Dec 2016.

[61] Nicodemus HF, Chaney RD, Herold R (1981). Hemodynamic effects of inotropes during hypothermia and rapid rewarming. Crit Care Med.

[62] Roscher R, Ingemansson R, Wetterberg T, Algotsson L, Sjöberg T, Steen S (1997). Contradictory effects of dopamine at 32 °C in pigs anesthetized with ketamine. Acta Anaesthesiol Scand.

[63] Filseth OM, How O-J, Kondratiev T, Gamst TM, Sager G, Tveita T (2012). Changes in cardiovascular effects of dopamine in response to graded hypothermia *in vivo**. Crit Care Med.

[64] Filseth OM, Fredriksen K, Gamst TM, Gilbert M, Hesselberg N, Naesheim T (2014). Veileder for håndtering av aksidentell hypotermi i Helse Nord.

[65] Polderman KH (2013). Of ions and temperature: the complicated interplay of temperature, fluids, and electrolytes on myocardial function. Crit Care.

[66] Pedersen TF, Thorbjørnsen ML, Klepstad P, Sunde K, Dale O (2007). [Therapeutic hypothermia--pharmacology and pathophysiology]. Tidsskr Nor Laegeforen.

[67] Tortorici MA, Kochanek PM, Poloyac SM (2007). Effects of hypothermia on drug disposition, metabolism, and response: a focus of hypothermia-mediated alterations on the cytochrome P450 enzyme system. Crit Care Med.

[68] Roizen MF, Weise V, Moss J, Kopin IJ (1975). Plasma catecholamines: arterial-venous differences and the influence of body temperature. Life Sci Elsevier.

[69] Kelly LA, Wu C, Butcher RW (1978). The escape of cyclic AMP from human diploid fibroblasts: general properties. J Cyclic Nucleotide Res.

[70] Schaible N, Han Y-S, Hoang T, Arteaga GM, Tveita T, Sieck GC. Hypothermia/rewarming disrupts excitation-contraction coupling in cardiomyocytes. Am J Physiol Heart Circ Physiol. 2016;310:H1533–40. 10.1152/ajpheart.00840.2015 PMC4935512 26993227

[71] Lewis ME, Al-Khalidi A-H, Townend JN, Coote J, Bonser RS (2002). The effects of hypothermia on human left ventricular contractile function during cardiac surgery. J Am Coll Cardiol.

[72] Eckmann DM, Bowers S, Stecker M, Cheung AT (2001). Hematocrit, volume expander, temperature, and shear rate effects on blood viscosity. Surv Anesthesiol.

[73] Dietrichs ES, Dietrichs E (2015). Neuroprotective effects of hypothermia. Tidsskr Nor Laegeforen.

[74] Bernard SA, Gray TW, Buist MD, Jones BM, Silvester W, Gutteridge G (2002). Treatment of comatose survivors of out-of-hospital cardiac arrest with induced hypothermia. N Engl J Med.

[75] Dietrichs ES, Kondratiev T, Tveita T (2014). Milrinone ameliorates cardiac mechanical dysfunction after hypothermia in an intact rat model. Cryobiology.

[76] Dietrichs ES, Håheim B, Kondratiev T, Sieck GC, Tveita T (2014). Cardiovascular effects of levosimendan during rewarming from hypothermia in rat. Cryobiology.

[77] Rungatscher A, Hallström S, Giacomazzi A, Linardi D, Milani E, Tessari M (2013). Role of calcium desensitization in the treatment of myocardial dysfunction after deep hypothermic circulatory arrest. Crit Care.

[78] Rungatscher A, Linardi D, Tessari M, Menon T, Luciani GB, Mazzucco A (2012). Levosimendan is superior to epinephrine in improving myocardial function after cardiopulmonary bypass with deep hypothermic circulatory arrest in rats. The journal of thoracic and cardiovascular surgery. J Thorac Cardiovasc Surg.

[79] Tveita T, Sieck GC (2012). Effects of milrinone on left ventricular cardiac function during cooling in an intact animal model. Cryobiology.

